# Gut microbiome partially mediates and coordinates the effects of genetics on anxiety-like behavior in Collaborative Cross mice

**DOI:** 10.1038/s41598-020-79538-x

**Published:** 2021-01-11

**Authors:** X. Jin, Y. Zhang, S. E. Celniker, Y. Xia, J.-H. Mao, A. M. Snijders, H. Chang

**Affiliations:** 1 Emergency Center, Zhongnan Hospital of Wuhan University, 169 Donghu Road, Wuhan, 430071 Hubei China; 2 Biological Systems and Engineering Division, Lawrence Berkeley National Laboratory, Berkeley, CA 94720 USA; 3 State Key Laboratory of Reproductive Medicine, Center for Global Health, School of Public Health, Nanjing Medical University, Nanjing, 211166 Jiangsu China; 4 Biological Systems and Engineering Division, Berkeley Biomedical Data Science Center, Lawrence Berkeley National Laboratory, Berkeley, CA 94720 USA

**Keywords:** Behavioural genetics, Microbiome

## Abstract

Growing evidence suggests that the gut microbiome (GM) plays a critical role in health and disease. However, the contribution of GM to psychiatric disorders, especially anxiety, remains unclear. We used the Collaborative Cross (CC) mouse population-based model to identify anxiety associated host genetic and GM factors. Anxiety-like behavior of 445 mice across 30 CC strains was measured using the light/dark box assay and documented by video. A custom tracking system was developed to quantify seven anxiety-related phenotypes based on video. Mice were assigned to a low or high anxiety group by consensus clustering using seven anxiety-related phenotypes. Genome-wide association analysis (GWAS) identified 141 genes (264 SNPs) significantly enriched for anxiety and depression related functions. In the same CC cohort, we measured GM composition and identified five families that differ between high and low anxiety mice. Anxiety level was predicted with 79% accuracy and an AUC of 0.81. Mediation analyses revealed that the genetic contribution to anxiety was partially mediated by the GM. Our findings indicate that GM partially mediates and coordinates the effects of genetics on anxiety.

## Introduction

Anxiety disorders are typically characterized by a heterogeneous cluster of common mental health symptomatology, including intense and sustained hyper-arousal, excessive fear and worry, frequently accompanied by somatic, behavioral, and cognitive distress responses^1^. Anxiety disorders are among the most frequently diagnosed adult psychiatric conditions, which often affect academic, professional performance and social and family interactions with lifetime consequences^2^.

Data from the Global Burden of Disease study in 2010 showed that anxiety disorders were the sixth most common cause of disability^3^, with global prevalence ranging from 3.8 to 25% across different countries^4^. In the United States, the estimated prevalence of adults with anxiety disorders is 18%, affecting approximately 40 million Americans with an annual cost of approximately $42.3 billion^5^. Meanwhile, in the European Union (EU), more than 60 million people experienced anxiety disorder(s) each year, making it the most common psychiatric concern in the EU^6^.

Family and twin studies suggest that familial and genetic components are among the best risk factors for anxiety disorders, although anxiety disorders are also influenced by environmental factors^7^. A recent large-scale GWAS among 12,655 individuals with various anxiety and stress-related diagnoses and 19,225 controls identified variants of the gene *Phosphodiesterase 4B (PDE4B)* encoding a protein that plays a role in signal transduction by regulating the cellular concentrations of cyclic nucleotides^8^. Altered activity of this protein has been associated with schizophrenia and bipolar affective disorder and with anxiety and stress-related disorder^9–11^. A meta-analysis of nine anxiety disorder GWAS identified an uncharacterized non-coding RNA locus (LOC152225) associated with lifetime diagnosis of anxiety spectrum disorders, and the gene *Calmodulin-Lysine N-Methyltransferase (CAMKMT)* associated with latent anxiety disorder factor-score model^7^. However, few other reproducible susceptibility genes related to anxiety disorders have been identified.

Recently, the gut microbiota have been identified to be associated with many neurodevelopmental and neurodegenerative diseases through the gut-brain axis^12^. Several lines of evidence indicated an important role for the gut microbiome in behavior (reviewed in)^13^. In the absence of microbes, the brain of mice was affected and colonization of animals with specific bacterial strains could alter behavior^14,15^. Furthermore, children exposed to infections exhibited an increased incidence of irritable bowel syndrome, which has been associated with psychological distress^16^. In mice, antibiotic induced dysbiosis caused depression, whereas in rats, temporal disruption of the gut microbiome by antibiotics early in life affected visceral pain in adulthood^17,18^. Moreover, anxiety and depression behaviors could be transferred by fecal matter transplantation. For example, the fecal microbiome from patients with major depressive disorder could induce depression-like behaviors after transplantation in germ-free mice^19^. These studies indicate that the gut microbiome plays an important role in anxiety and depression-like disorders and highlight that this effect appears to be dependent on host genetics and environment^20,21^. Modulating the gut microbiota and subsequently regulating the gut-brain axis are currently being explored in an effort to improve mental health. So far, interventions using probiotics and prebiotics have shown promising results in terms of efficacy^22^. Chronic treatment with *Lactobacillus rhamnosus JB-1* reduced anxiety-like behaviors and altered GABAB1b expression in the brain of mice^23^. *Bifidobacterium longum NCC3001* normalized anxiety-like behavior and expression of hippocampal brain derived neurotrophic factor (BDNF) in mice with infectious colitis^24^. However, the mechanisms behind the regulation and interventions of gut-brain communication and function remains largely unexplored.

In this study, we used the Collaborative Cross (CC) mouse population-based model to measure anxiety-like behavior by the widely used light-dark test and to investigate the complex interaction between genetics, the gut microbiome and anxiety related phenotypes. The CC mouse model represents a genetically heterogeneous population with evenly distributed allele variation, and an allele frequency distribution similar to that of the human population^25,26^. This model has been used to study for example individual variation in motor performance^27^, gut microbiome composition^28^, cancer susceptibility^29^, memory performance^30^ and viral pathogenesis^31^. This study provides new evidence for a complex interaction between host genetics, the gut microbiota and anxiety, and shows that the effect of genetics on anxiety can at least partially be mediated by the microbiome.

## Results

### Computational pipeline for mouse behavior characterization

**Figure 1:**
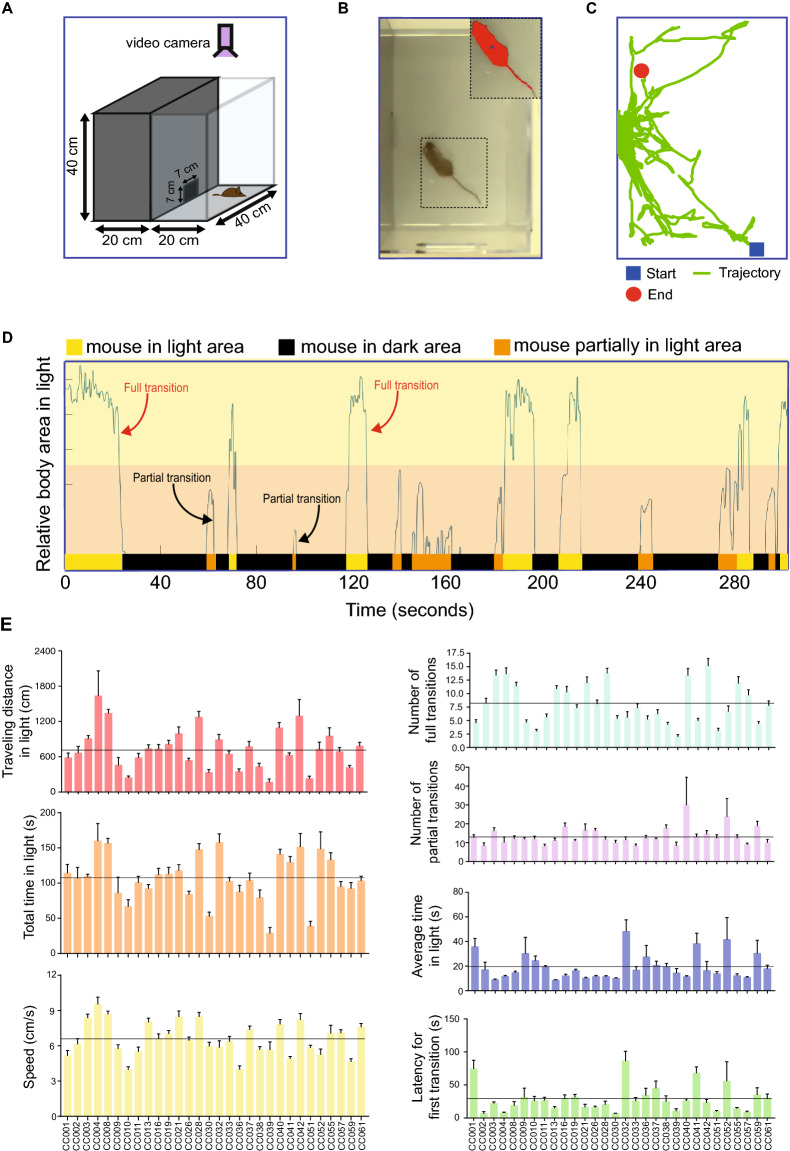
Measurement of anxiety-related phenotypes in Collaborative Cross mice using the light-dark box. (**A**) Dimensions of the light-dark box. (**B**) Mouse behavior video tracking in the light compartment. (**C**) Trajectory of a single mouse in the light compartment. The trajectory is indicated with a green line. The start and end points are indicated with a blue box and red circle, respectively. (**D**) Representative profile of mouse behavior in the light-dark box during the 300 second assay time. Transitions between the light and dark compartments was determined by measuring the relative body area in the light compartment. The bar at the bottom of the profile indicates the presence of the mouse in the dark area (black), the light area (yellow) or in between the light and dark compartments (orange). (**E**) Average measurements of seven anxiety-related phenotypes across CC strains. Bars indicate the mean measurement and error bars indicate standard deviation. The horizontal line indicates the mean value across all strains.

We used the light-dark box to assess the variation in anxiety-like behavior in 445 mice (228 female; 217 male) representing 30 CC strains with video recording of the light compartment. The number of mice for each strain ranged from 7 to 25 (Table S1). A computational pipeline was developed to quantify anxiety levels by tracking mouse location (Fig. 1A–D). Seven anxiety-related phenotypes were extracted from each video file: number of full and partial transitions between light and dark, speed, distance traveled and total time spent in the light compartment, average time spent in light for each transition and latency to first transition into the dark compartment (Table 1). Correlation analysis between anxiety related phenotypes revealed several positive correlations including for example between total time spent in the light compartment and distance traveled in the light compartment (R=0.86; FDR<0.0001) and average speed and number of full transitions (R=0.76; FDR<0.0001) (Fig. S1). Negative correlations were observed between average time in light and number of full transitions (R=-0.56; FDR<0.0001) and average speed (R=-0.49; FDR<0.0001) (Fig. S1).

**Table 1:** Description of anxiety-related phenotypes.

Anxiety-related phenotype	Phenotype description
Traveling distance in light	Total distance travelled in light area of light/dark box
Number of full translations	Number of whole-body transitions from light area to dark area of light/dark box
Total time in light	Total time spent in light area of light/dark box
Number of partial translations	Number of partial-body transitions from dark to light and then back to dark area of light/dark box
Average speed	Average speed in light area of light/dark box
Average time in light	Average time spent in light area of light/dark box
Latency time for first transition	Time spent in light before first transition from light to dark area of light/dark box

### Anxiety related behavior phenotypes vary across CC strains

The travelling distance in the light compartment ranged from 1649mm (CC039) to 16311mm (CC004), the number of full transitions ranged from 2 (CC039) to 15 (CC042), the number of partial transitions ranged from 8 (CC011) to 30 (CC040), the total time spent in the light compartment ranged from 28.58s (CC039) to 159.84s (CC004), the average speed in the light compartment ranged from 39.54mm/s (CC010) to 95.24mm/s (CC004), the latency time for the first transition ranged from 6.44s (CC030) to 86.12s (CC032), and the average time in light ranged from 6.44s (CC030) to 86.18s (CC032) (Fig. 1E, Table S1). No significant difference was observed between male and female mice from any of the CC strains (adjusted p>0.05; Fig. S2). We observed significant variation in these seven anxiety-related phenotypes across different CC strains, strongly suggesting that host genetics influences anxiety.

### Anxiety level assessment based on seven anxiety related phenotypes

**Figure 2:**
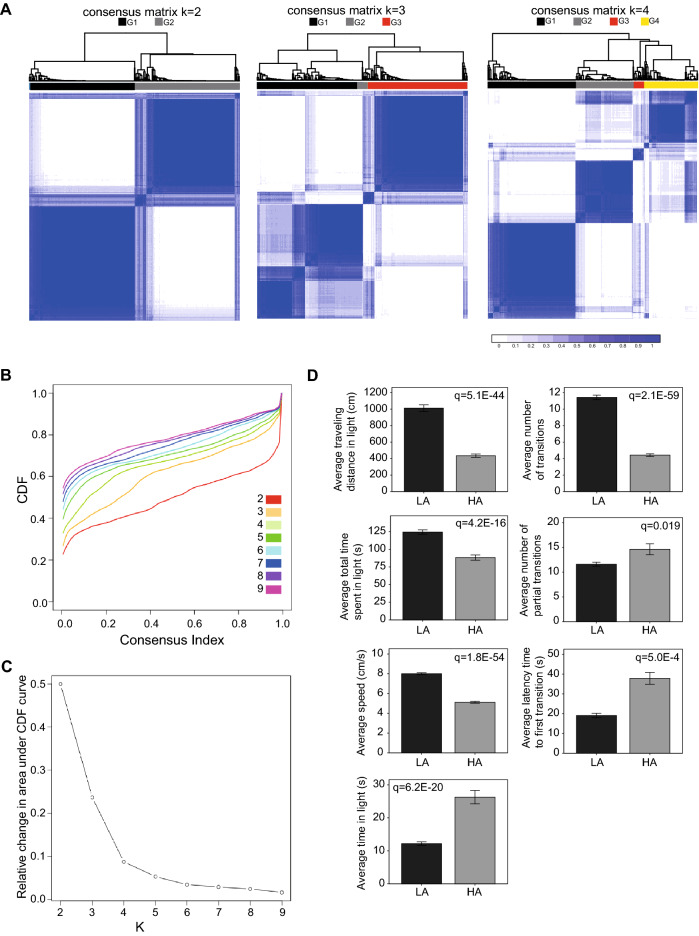
Classification of CC mice into low and high anxiety based on seven anxiety-related phenotypes. (**A**) Consensus classification heatmaps based on two, three or four clusters. A relative stable partitioning of the samples is seen at K=2. The blue color in the clustering diagram refers to the consistency by which each sample is assigned to a specific cluster (dark blue indicates 100% confidence in the assigned class). (**B**) Cumulative Distribution Function (CDF) plot of consensus membership values for solutions with two to nine clusters. (**C**) Delta area curve of consensus clustering for solutions with two to nine clusters. (**D**) Difference in seven anxiety-related phenotypes between mice assigned to the low anxiety (LA) group and mice assigned to the high anxiety (HA) group. P-values were obtained by Mann-Whitney test and corrected for multiple comparisons by Benjamini-Hochberg.

In order to classify mice into different anxiety level, we combined the anxiety-related phenotypes of our mouse cohort and employed consensus clustering to obtain anxiety-related subgroups using different numbers of clusters (K = 2, 3 and 4; Fig. 2A). Consensus clustering visualizes the consistency by which each sample is assigned to a specific cluster. The cumulative distribution function (CDF) and change in the area under the curve for CDF at different values of K suggests maximum stability at K=4 (Fig. 2B,C). However, visual inspection of the consensus matrices, showed that dividing the mouse cohort into two subgroups (K= 2) corresponding to low and high anxiety levels resulted in the most consistent matrix (Fig. 2A). Based on the result of the consensus clustering, we have divided the mouse cohort into two groups: high anxiety (HA) and low anxiety (LA). The seven anxiety-related phenotypes were significantly different between the two groups (Mann-Whitney U-test; FDR < 0.01; Fig. 2D), and multivariate logistic regression analysis indicates that five out of seven anxiety-related phenotypes independently contribute to the anxiety classification (Table S2; p<0.05). All mice from strains CC013, CC004 and CC008 were classified as LA, whereas all mice from strains CC010, CC038 and CC051 were classified as HA (Fig. S3). For each of the remaining strains, individual mice were assigned to either LA or HA suggesting incomplete penetrance of the anxiety-like phenotype. Data from all mice was included in downstream analyses.

### Genome-wide associations between anxiety and genetics

**Figure 3:**
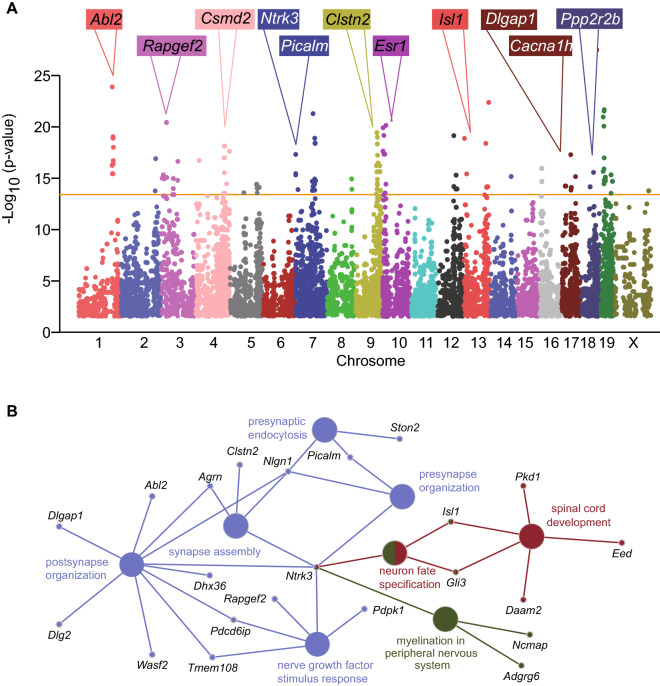
Identification of genetic variations and candidate genes associated with anxiety in CC mice. (**A**) Manhattan plot of the genome-wide association analysis for anxiety in CC mice. The –log_10_(P-value) is shown for 70,273 SNPs ordered based on genomic position. The horizontal orange line (–log_10_(P-value)=13) indicates the significance threshold. (**B**) Gene Ontology (GO) analysis of genes identified in QTL associated with anxiety in Fig. 4A. Candidate genes are significantly enriched for biological processes related to neuronal function (P<0.05).

To investigate the contribution of genetic variation to anxiety-like behavior, genome-wide association study (GWAS) analysis was performed with 70,273 SNPs across 30 CC strains comparing HA with LA mice. We identified 264 SNPs significantly associated with anxiety (p < 1.00E-13) corresponding to 141 named genes (Fig. 3A; Tables S3 and S4). Gene Ontology analysis revealed that 141 genes were significantly enriched in biological processes related to neuronal function including synapse assembly, neuron fate specification and presynaptic endocytosis (p<0.05; Fig. 3B). Our screen identified 62 genes known to be associated with anxiety, behavioral alterations and neurodevelopment of which 40 genes show expression in the brain based on in situ hybridization data from the Mouse Brain Atlas (Allen Brain Atlas) (Table S4). For instance, allele variants of *NTRK3*, *PPP2R2B*, and *ESR1* were associated with anxiety in humans^32–34^, knock-out mice of *Cacna1h*, *Rapgef2*, *Clstn2*, and *Tnr* exhibited abnormal anxiety-like behaviors^35–38^, and *Isl1*, *Abl2*, *Dlgap1* and *Csmd2* knock-out mice showed alterations in neurodevelopment and behavior^39–42^. In addition to these 62 known genes, our screen identified 79 genes not previously associated with anxiety, which includes 38 genes that show expression in the brain based on *in situ* hybridization data from the Mouse Brain Atlas (Allen Brain Atlas) (Table S4). The spatial gene expression data suggests that these 38 genes may play a role in anxiety.

### Associations between microbiome and anxiety-like behavior

**Figure 4:**
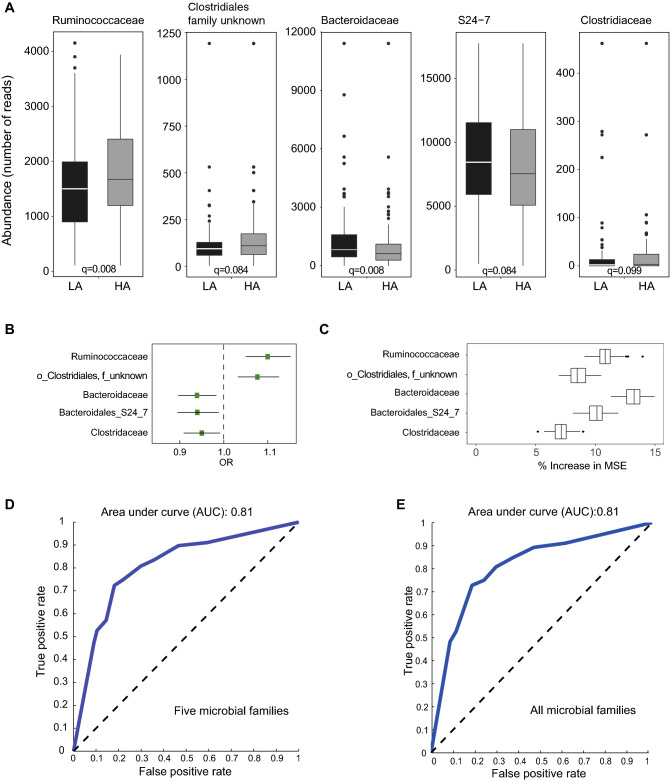
Association of gut microbiome with anxiety. (**A**) Family-level microbial abundance level differences between mice classified in LA and HA. P-values were obtained by Mann-Whitney test and corrected for multiple comparisons by Benjamini-Hochberg. (**B**) Relative risk score (OR: odds ratio) for microbial families associated with anxiety was calculated by logistic regression (FDR < 0.05). (**C**) Random forest analysis to assess the relative contribution of microbial families associated with anxiety. Significantly associations are indicated (FDR < 0.05). (**D**, **E**) Receiver operant characteristic (ROC) curves based on the abundance level of five microbial families associated with anxiety (**D**) and all microbial families (**E**). The accuracy of predicting anxiety levels was estimated by measuring the area under the ROC curve (AUC).

To investigate the association between specific microbes and anxiety-like behavior, we collected fecal samples from 30 CC strains for 16S ribosomal RNA profiling. Sequence reads were mapped to 5761 OTUs corresponding to 71 bacterial families^30^. We identified five families with significantly different abundance levels between high anxiety and low anxiety groups (Mann-Whitney U-test; FDR < 0.1; Fig. 4A). We observed higher relative abundances of *Ruminococcaceae, Clostridiaceae, Clostridiales_family unknown,* and lower relative abundances of B*acteroidaceae* and *Bacteroidales_S24-7* in HA mice compared to LA mice (Fig. 4A). Logistic regression confirmed that these families were significantly correlated with anxiety (FDR < 0.01; Fig. 4B). Abundance levels of *Ruminococcaceae* and *Clostridiales_family unknown* were positively correlated with anxiety level, while the abundance of *Bacteroidaceae, Clostridiaceae* and *Bacteroidales_S24-7* were negatively correlated with anxiety level (Fig. 4B; FDR < 0.05). At the OTU level we found that the abundance level of 327 OTUs was significantly different between the high anxiety and low anxiety groups (Mann-Whitney U-test; FDR < 0.05; Table S5). For example, we observed decreased abundance of bacteria from the genus *Bacteroides* (family *Bacteroidaceae*) and increased abundance of several genera from the family *Ruminococcaeae* including *Ruminiclostridium, Ruminococcaceae_UCG-014* and *Oscillibacter* in high anxiety compared to low anxiety mice.

To determine the importance for each of the five families related to anxiety, we performed random forest classification analysis and show that *Bacteroidaceae* and *Ruminococcaceae* contribute most to the anxiety phenotype (Fig. 4C). Furthermore, random forest classification based on the five families related to anxiety level (i.e., HA and LA) resulted in a predictive accuracy of 79% with an AUC around 0.81 (Fig. 4D), where the predictive power is statistically identical with the one derived from all microbiome features (Fig. 4E). Our results demonstrate an association between microbiome features and anxiety-like behavior.

### Gut microbiome partially mediates the effects of genetics on anxiety-like behavior

**Figure 5:**
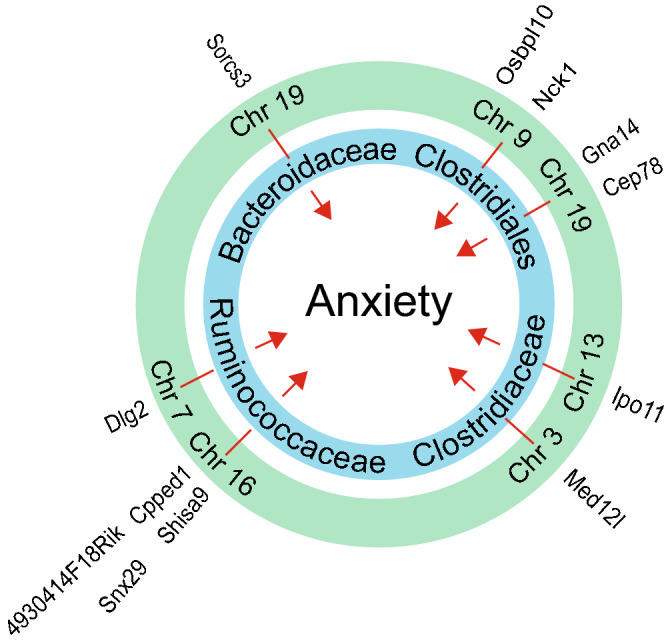
Microbial families mediate the effect of host genetics on anxiety. Four microbial families were identified as mediators between genetic variants and anxiety. Green ring indicates chromosomal locations associated with anxiety and abundance levels of microbial families indicated in the blue ring. Candidate genes within each genetic locus are listed on the outside of the green ring.

We then performed mediation analysis to investigate whether genetic variants indirectly contribute to anxiety-like behavior by controlling the abundance of the five families associated with anxiety. Mediation analysis is a statistical model to determine whether the relationship between two variables (genetic variants and anxiety) is mediated through a third variable (gut microbiome). We first identified 12,368 genetic variants significantly (Mann-Whitney U-test; p<1E-09) associated with any of the five microbial families. We then selected only genetic variants significantly associated with both the abundance of any of the five microbial families and anxiety-like behavior. Mediation analysis revealed that *Ruminococcaceae*, *Clostridiaceae, Bacteroidaceae* and *Clostridiales* (family unknown) function as mediators for the effect of 17 SNPs within 7 genetic loci on anxiety (Fig. 5, Table S6; FDR <0.05). These analyses indicated that the effect of genetic variations on anxiety is at least partially mediated by the gut microbiota, and therefore suggested that the gut-brain axis plays important roles in anxiety disorders.

### Anxiety related genes show significant overlap with human GWAS for psychiatric conditions

**Figure 6:**
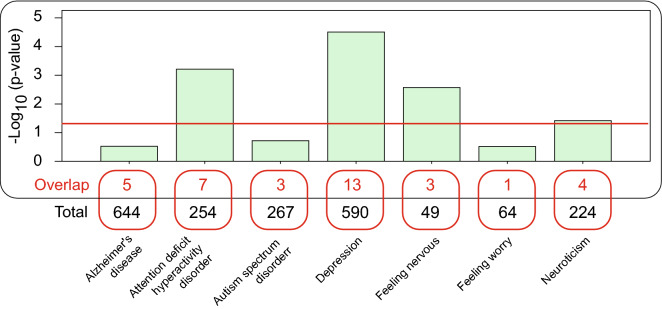
Overlap between murine candidate genes associated with anxiety and genes associated with human anxiety-related phenotypes and neuropsychological disorders. The overlap between mouse anxiety associated genes and genes associated with Alzheimer’s disease, attention deficit hyperactivity disorder, austism spectrum disorder, depression, feeling nervous, feeling worry and neuroticism. The red line indicates significant overlap (p<0.05).

To discover candidate genes with potential impact on human anxiety, we compared anxiety-related mouse genes (141 genes) to a compiled list of human genes associated with seven psychiatric conditions that have been previously identified by human GWAS (Table S7). We observed significant overlap between the mouse anxiety associated genes and genes associated with attention deficit hyperactivity disorder, depression, feeling nervous, and neuroticism (Fig. 6; p<0.05). We found that 25 out of our 141 anxiety-related mouse genes were associated with one or more human GWAS for psychiatric conditions. Interestingly, *STAG1* and *SORCS3* were both found in four phenotypes. *STAG1* was associated with autism spectrum disorder, feeling nervous, feeling worry and neuroticism, whereas *SORCS3* was associated with Alzheimer, depression, feeling nervous and neuroticism. We thus conclude that the candidate mouse genes identified in this anxiety associated study exhibit significant relevance with human psychiatric conditions.

## Discussion

In our study, a systematic genome and metagenome analysis on anxiety-like behavior was performed on 445 mice across 30 genetically defined CC strains to identify the effects of host genetics and gut microbiota and their interaction on anxiety. Our findings have the potential to provide insights on the mechanisms of host–microbe interactions related to anxiety. We further demonstrate that genetic effects on anxiety are partially mediated through modulating the abundance of specific gut microbes, suggesting links between host genetics and anxiety via intestinal health.

We developed a pipeline, integrating multiple phenotypes, for assessing anxiety-like behavior in mice. In previous mouse anxiety studies^43,44^, individual behavior phenotypes (e.g. percentage of time spent in light chamber and number of full transitions) are frequently independently utilized for the determination of the level of anxiety with manually defined thresholds. However, there are a number of challenges with this traditional approach: (I) threshold selection for each anxiety-related behavior phenotype may be subjective and (II) integration of multiple different behavior phenotypes may be ad-hoc. Furthermore, anxiety assessment in clinical practice for human patients (e.g., The Human Concern Scale, commonly used by State-Trait Anxiety Inventory, Beck Anxiety Inventory, and Hospital Anxiety and Depression Scale-Anxiety) typically involves the consideration and aggregation of multiple factors^45^. Therefore, systematic characterization of mouse behavior with multivariate capabilities may more accurately define anxiety level subgroups. Here, we integrated seven anxiety-related behavior phenotypes and utilized consensus clustering for the automatic identification of anxiety level subgroups.

Using the CC mouse population model with diverse and reproducible genetic backgrounds, we identified 264 SNPs corresponding to 141 known that are significantly associated with anxiety-like behavior. A number of genes identified in our study have been linked to anxiety-like phenotypes in transgenic animal models and confirm that population-based studies using CC mice are a powerful and unbiased approach to identify candidate genes associated with specific phenotypes. For example, *Ntrk3*, *Tnr*, *Cacna1h*, *Clstn2*, and *Rapgef2* were found to be involved in pathological processes of anxiety. The mRNA expression of Neurotrophic Receptor Tyrosine Kinase 3 (*Ntrk3*) decreased in central amygdala nucleus of young primates with high anxious temperament^46^. Overexpression of NTRK3's endogenous ligand (*Ntf3*) in the dorsal amygdala resulted in reduced anxious temperament and altered function in the anxious temperament neural circuit, implicating the role of neurotrophin-3/NTRK3 signaling in mediating primate anxiety^47^. *Tnr* knockout mice displayed decreased motivation to explore and an increased anxiety, which was more easily influenced by environmental factors^38^. Anxiety analysis in the BXD recombinant inbred mouse population also identified *Tnr* and subsequent systems genetic analysis showed that *Tnr* was co-expressed with genes related to psychiatric disorders^48^. Overexpression of *Cacna1h* induced anxiety and genetic ablation of the *Cacna1h* gene results in an anxiety-like phenotype in mice, suggesting normal *Cacna1h* state is crucial to anxiety and both activation and inhibition of these channels in stressful condition may produce anxiety^35^. *Clstn2* knockout mice displayed high exploration and hyperactivity affecting anxiety parameters^37^. *Rapgef2* knockout mice exhibited hyperlocomotion phenotypes and decreased anxiety-like behavior^36^.

In addition to genes already associated with anxiety, our study identified a number of genes not previously associated with anxiety. A role for *Abl2*, *Csmd2*, *Dlgap1*, and *Isl1* in neurodevelopment and psychiatric and neurodegenerative diseases have been reported in previous studies and our association analysis suggests a role in anxiety. Activation of Abl2/Arg kinase can alleviate corticosteroid-induced dendrite loss and behavioral deficiencies whereas *Arg* knockout mice exhibit synapse and dendrite loss and behavioral deficiencies^40^. Knock-down of *Csmd2* results in reduced filopodia density in immature developing neurons and reduced dendritic spine density and dendrite complexity, implicating its association with certain psychiatric disorders^42^. *Dlgap1* knockout mice exhibit post-synaptic density (PSD) disruption and reduced sociability, consistent with reports of *Dlgap1* variants in schizophrenia and autism spectrum disorder (ASD)^41^. Conditional deletion of *Isl1* using a *Six3-cre* transgene results in an early and persistent defect in cholinergic neuron differentiation and these dysfunctions have been implicated in various psychiatric and neurodegenerative diseases^39^. The comparison of our anxiety-related genes with human GWAS for neurological conditions identified *Stag1* and *Sorcs3* associated with four conditions including “Alzheimer's disease”, “autism spectrum disorder, “depression”, “feeling nervous”, “feeling worry”, and “neuroticism”. The roles of *Stag1* and *Sorcs3* in anxiety were not reported before, but the human GWAS data and our mice GWAS data imply that there could be a connection between these genes and anxiety.

The gut–brain axis plays important roles in neuropsychiatric disorders. Bidirectional interactions between the central nervous system and gut microbiota are maintained by different pathways including: direct activation of neuronal pathways, microbial metabolism of nutrients and production of circulating mediators, and immune activation and circulating inflammatory mediators. Our results showed that abundance of *Ruminococcaceae* was significantly higher in HA than in LA mice, and was positively correlated with the level of anxiety. Kang et al also showed that abundance of *Ruminococcaceae* correlated negatively with “percent of time in light” (i.e. lower *Ruminococcaceae* levels correlated with less anxiety)^49^. This study suggests that specific gut microbes could be used as a biomarker for anxiety or cognition and perhaps even targeted for therapy^49^. In a separate study, the levels of depression, anxiety, and eating disorder psychopathology at an inpatient admission were associated with the composition and diversity of the intestinal microbiota^50^. Significant changes in the composition of the intestinal microbiota were seen in patients with anorexia nervosa during re-nourishment, particularly among genera falling in the family *Ruminococcaceae*. Furthermore, in a study of psychological distress in patients with irritable bowel syndrome identified that patients with anxiety were characterized by elevated *Bacteroidaceae*^51^. However, not all literature is consistent in terms of abundance levels of *Ruminococcaceae* and anxiety. For example, anxiety and depression were associated with decreases in OTUs belong to the family *Ruminococcaceae*^52^. Also, abundance levels of *Ruminococcaceae_UCG-014* correlated negatively with anxiety severity and positively with anxiety reduction^53^. To date, there appears to be no known mechanism regarding the role of *Ruminococcaceae* in anxiety, and further studies are needed to explore this. Studies in mice and humans have shown that the abundance level of pathogenic bacteria can increase anxiety-like behavior^54,55^, which could be due to their ability to produce exotoxins and promote conditions favoring inflammation. Our mediation analysis indicated that the effect of seven genetic loci could affect anxiety by altering abundance levels of *Ruminococcaceae, Bacteroidaceae and Clostridiaceae*. For example, our study suggests that *Sorcs3* regulates anxiety by modulating abundance of *Bacteroidaceae*. Interestingly, genetic variants in *Sorcs3* have previously been associated with abundance levels of *Peptoniphilus* in the nasopharynx^56^. These findings suggest that the association of SORCS3 with Alzheimer, depression, feeling nervous and neuroticism could be mediated by changes in the host microbiome.

The pathogenesis of anxiety disorders is complex, and involves intricate interactions between biological factors, environmental influences and psychological mechanisms. Even though we have found genetic and microbiome influences on anxiety disorders the genetic underpinnings of anxiety remains poorly understood. Together, our results suggest a complex genetic-microbiome interplay in the modulation of mouse anxiety. This study lays the foundation for future research to evaluate treatments for anxiety taking into account both host genome and microbiome.

## Materials and methods

### Mice maintenance

CC mouse strains were purchased from the Systems Genetics Core Facility at the University of North Carolina (UNC)^57,58^. The CC represents a large panel of multiparental recombinant inbred mouse lines for analysis of phenotype-genotype associations of genetic traits. Mice were acclimated at Lawrence Berkeley National Laboratory (LBNL) for two weeks prior to behavior testing. Mice were maintained on PicoLab Rodent Diet 20, raised in standard micro-isolator cages on corncob bedding with crinkle cut paper strand enrichment. Animals were maintained in a light-controlled room (12:12 h light/dark cycle). Light/dark box test was conducted at 12 weeks of age. The study followed the National Institutes of Health's guidelines for the Care and Use of Laboratory Animals. The Animal Welfare and Research Committee of the Lawrence Berkeley National Laboratory approved all animal procedures (Protocol File Number 271004). The number of mice for each of 30 strains used in this study is shown in Table S1.

### Light/dark test and mouse behavior characterization

We performed a light/dark (LD) behavioral test commonly used to study anxiety-like behavior in mice^59^. The light/dark test is based on rodents' natural aversion to bright areas and their spontaneous exploratory behavior. The device consists of two compartments each a polyvinylchloride box (20 cm × 40 cm × 40 cm) covered with plexiglas, one of the boxes is black and the other is transparent and illuminated. A small opening (7 cm ×7 cm) connects the two compartments. The light/white compartment was illuminated by a light from the ceiling (~350 lx at the floor of light/dark apparatus), while the black/dark compartment was not illuminated. Individual mice were placed into the light/white compartment, and then allowed to explore the enclosure freely for 5 minutes. All testing was conducted during the animal’s light cycle. A video camera recorded the light compartment of the apparatus. Between consecutive tests, the instrument was cleaned with water. After the LD test, we constructed a computational pipeline to systematically evaluate mouse behavior following three steps: (1) mouse tracking, (2) behavior profiling and (3) feature extraction (Fig. 1A–E). The first two steps resulted in the mouse behavior profile, which characterizes the dynamic mouse size in the light chamber across time and provides a visualization tool for phenotype definition, extraction and quality control. Finally, seven anxiety-related phenotypes were quantified (Table 1).

### Anxiety level characterization

Consensus clustering (ConsensusClusterPlus package v1.50 in *R*) was performed using all mice in our study to determine behavior subgroups that correlate with different anxiety levels based on seven anxiety-related phenotypes, where 80% of the samples were bootstrapped 100 times. Euclidian distance was adopted for similarity measurement, and k-mean clustering was used as the clustering algorithm. After consensus clustering, the number of clusters was determined by the consistency of clusters, and the cumulative distribution information among different numbers of clusters. Finally, based on K=2 clusters, we assigned each mouse to either high anxiety (HA) or low anxiety (LA) groups (Table S1). Data from all mice was used for genetic association, microbiome association and mediation analysis.

### Fecal sample collection and microbiome analysis

Fecal samples were collected from individual cages 16 h after a cage change as reported previously^14,28^ 24 hours prior to the light/dark test. At least four independent cages were sampled for each CC strain. After collection, fecal samples were stored at -80°C until microbial analysis. We extracted genomic DNA from the homogenized fecal samples using the PowerSoil DNA Isolation Kit (http://www.mobio.com/) according to the manufacturer's instructions. PCR amplification of the V4 region of the 16S rRNA gene was performed using modern primers^60^. Amplicons were sequenced on an Illumina MiSeq using paired, 250 base-pair reads, according to the manufacturer's instructions and analyzed as previously described^30^. Animals were group housed whenever possible and individual mice were assigned the microbiome profile from their respective cage.

### QTL analysis of anxiety

Genotype data of 134,593 SNPs was obtained from the UNC Systems Genetics Core website (http://csbio.unc.edu/CCstatus/index.py). SNPs were filtered based on minor allele frequency ≥3 out of the 30 CC strains, leaving 70,273 SNPs. At each SNP, anxiety level (i.e., sub-group assignment) for all CC mice were assigned to their respective alleles. The Chi-square test was used to test the significance of associations between anxiety level (high anxiety or low anxiety based on consensus clustering) and allele classes at each SNP. SNPs with a p-value less than 1.00E-13 were selected. Putative candidate genes were defined as those genes (gencode.vM741) containing a significant SNP within the boundaries of the gene sequence. Gene Ontology biological annotations of putative genes were determined using ClueGO and visualized in Cytoscape. Human GWAS data was downloaded on 09/13/2019 from https://www.ebi.ac.uk/gwas/. The significance of overlap between mouse and human candidate genes was calculated based on the hypergeometric distribution^61^.

### Association between microbiome and anxiety levels

Mann-Whitney U Test (Matlab 2012b; Statistics Toolbox version 8.1) was utilized to identify microbiome features that have significantly different abundance (FDR < 0.1) between anxiety levels. Pre-selected microbiome features were correlated with anxiety levels using logistic regression (R 3.6.0; stats Package 3.6.1) (FDR < 0.01). To evaluate the effectiveness of significant microbiome features for the prediction of anxiety levels, random forest classification model (Matlab Random Forest Package version 4.6-14) was used with 100 cross-validation iterations, where, in each iteration, the number of trees was experimentally optimized to be 1000, 90% of samples were randomly selected for training and the rest 10% samples were used for testing.

### Mediation analysis

To explore whether microbiomes were mediators of the relation between genotype and anxiety, mediation analysis was performed (R 3.6.0; Mediation Package 4.4.7), to evaluate the mediation effect of mouse gut microbiome between genotype and anxiety levels. Specifically, we used the genotype as the treatment variable, and the gut microbiome as the mediator; and adopted the linear regression fit with least squares and the probit regression for the mediator and outcome models, respectively. The mediation effect and direct effect were then estimated with bootstrapping strategy using 1000 iterations. Details (including R script and sample data) about the mediation analysis can be found on: https://bbds.lbl.gov/release/microbiome-gentics-anxiety-10.

### Statistics

All statistical analyses were performed using R software^62^ (version 3.6.0) and Matlab (version 2012b), and the following packages were used: Mediation (R, version 4.4.7), Stats (R, version 3.6.1), Random Forest (Matlab, version 4.6-14), Statistics Toolbox (Matlab, version 8.1).

## Supplementary information

Supplementary Information 1.

Supplementary Information 2.

Supplementary Information 3.

Supplementary Information 4.

Supplementary Information 5.

Supplementary Information 6.

Supplementary Information 7.

Supplementary Information 8.

Supplementary Information 9.

Supplementary Information 10.

Supplementary Information 11.

## Data Availability

All data needed to evaluate the conclusions in the paper are presented in the paper and/or the Supplementary Materials. Additional data related to this paper may be requested from the authors. Mouse gut microbiome 16S rRNA gene sequence data is available on OSF (https://osf.io/jbt5g/).

## References

[1] Olthuis, J. V., Watt, M. C., Bailey, K., Hayden, J. A. & Stewart, S. H. Therapist-supported Internet cognitive behavioural therapy for anxiety disorders in adults. *Cochrane Database of Syst. Rev.* **3**, Cd011565. 10.1002/14651858.CD011565.pub2 (2016). 10.1002/14651858.CD011565.pub2 PMC7077612 26968204

[2] Craske MG (2017). Anxiety disorders. Nat. Rev. Dis. Prim..

[3] Rehm J, Shield KD (2019). Global burden of disease and the impact of mental and addictive disorders. Curr. Psychiatry Rep..

[4] Remes O, Brayne C, van der Linde R, Lafortune L (2016). A systematic review of reviews on the prevalence of anxiety disorders in adult populations. Brain Behav..

[5] GBD 2017 Disease and Injury Incidence and Prevalence Collaborators SL, A. D., Abate KH, Abay SM, Abbafati C, Abbasi N, et al. Global, regional, and national incidence, prevalence, and years lived with disability for 354 diseases and injuries for 195 countries and territories, 1990–2017: a systematic analysis for the Global Burden of Disease Study 2017. *Lancet (London, England)* **392**, 1789-1858. 10.1016/s0140-6736(18)32279-7 (2018). 10.1016/S0140-6736(18)32279-7 PMC6227754 30496104

[6] Wittchen HU (2011). The size and burden of mental disorders and other disorders of the brain in Europe 2010. Eur. Neuropsychopharmacol. J. Eur. Coll. Neuropsychopharmacol..

[7] Bartlett AA, Singh R, Hunter RG (2017). Anxiety and epigenetics. Adv. Exp. Med. Biol..

[8] Meier SM (2019). Genetic variants associated with anxiety and stress-related disorders: a genome-wide association study and mouse-model study. JAMA Psychiatry..

[9] McDonald ML, MacMullen C, Liu DJ, Leal SM, Davis RL (2012). Genetic association of cyclic AMP signaling genes with bipolar disorder. Transl. Psychiatry.

[10] Meier SM (2019). Genetic variants associated with anxiety and stress-related disorders: a genome-wide association study and mouse-model study. JAMA Psychiatry..

[11] Pickard BS (2007). The PDE4B gene confers sex-specific protection against schizophrenia. Psychiatry Genet.

[12] Liang S, Wu X, Jin F (2018). Gut-brain psychology: rethinking psychology from the microbiota-gut-brain axis. Front. Integr. Neurosci..

[13] Cryan JF (2019). The microbiota-gut-brain axis. Physiol. Rev..

[14] Mao JH (2020). Genetic and metabolic links between the murine microbiome and memory. Microbiome.

[15] Diaz Heijtz, R. *et al.* Normal gut microbiota modulates brain development and behavior. *Proc. Natl. Acad. Sci. USA* **108**, 3047-3052. 10.1073/pnas.1010529108 (2011). 10.1073/pnas.1010529108 PMC3041077 21282636

[16] Thabane M (2010). An outbreak of acute bacterial gastroenteritis is associated with an increased incidence of irritable bowel syndrome in children. Am. J. Gastroenterol..

[17] O'Mahony SM (2014). Disturbance of the gut microbiota in early-life selectively affects visceral pain in adulthood without impacting cognitive or anxiety-related behaviors in male rats. Neuroscience.

[18] Guida F (2018). Antibiotic-induced microbiota perturbation causes gut endocannabinoidome changes, hippocampal neuroglial reorganization and depression in mice. Brain Behav. Immunol..

[19] Zheng P (2016). Gut microbiome remodeling induces depressive-like behaviors through a pathway mediated by the host's metabolism. Mol. Psychiatry.

[20] Neufeld KM, Kang N, Bienenstock J, Foster JA (2011). Reduced anxiety-like behavior and central neurochemical change in germ-free mice. Neurogastroenterol. Motil..

[21] Nishino R (2013). Commensal microbiota modulate murine behaviors in a strictly contamination-free environment confirmed by culture-based methods. Neurogastroenterol. Motil..

[22] Burokas A (2017). Targeting the microbiota-gut-brain axis: prebiotics have anxiolytic and antidepressant-like effects and reverse the impact of chronic stress in mice. Biol. Psychiatry.

[23] Bravo JA (2011). Ingestion of Lactobacillus strain regulates emotional behavior and central GABA receptor expression in a mouse via the vagus nerve. Proc. Natl. Acad. Sci. USA.

[24] Bercik P (2011). The anxiolytic effect of Bifidobacterium longum NCC3001 involves vagal pathways for gut-brain communication. Neurogastroenterol. Motil. Off. J. Eur. Gastrointest. Motil. Soc..

[25] Threadgill DW, Miller DR, Churchill GA, de Villena FP (2011). The collaborative cross: a recombinant inbred mouse population for the systems genetic era. ILAR J.

[26] implications for QTL discovery and systems genetics (2007). Roberts, A., Pardo-Manuel de Villena, F., Wang, W., McMillan, L. & Threadgill, D. W. The polymorphism architecture of mouse genetic resources elucidated using genome-wide resequencing data. Mamm. Genome.

[27] Mao JH (2015). Identification of genetic factors that modify motor performance and body weight using Collaborative Cross mice. Sci. Rep..

[28] Snijders AM (2016). Influence of early life exposure, host genetics and diet on the mouse gut microbiome and metabolome. Nat. Microbiol..

[29] Wang P (2019). Diverse tumour susceptibility in Collaborative Cross mice: identification of a new mouse model for human gastric tumourigenesis. Gut.

[30] Mao JH (2020). Genetic and metabolic links between the murine microbiome and memory. Microbiome.

[31] Ferris MT (2013). Modeling host genetic regulation of influenza pathogenesis in the collaborative cross. PLoS Pathog..

[32] Muiños-Gimeno M (2009). Allele variants in functional MicroRNA target sites of the neurotrophin-3 receptor gene (NTRK3) as susceptibility factors for anxiety disorders. Hum. Mutat..

[33] O'Hearn E, Holmes SE, Calvert PC, Ross CA, Margolis RL (2001). SCA-12: Tremor with cerebellar and cortical atrophy is associated with a CAG repeat expansion. Neurology.

[34] Scariot R (2019). Association between gender, estrogen receptors genes and anxiety levels in patients undergoing orthognathic surgery. J. Cranio-maxillo-fac. Surg. Off. Publ. Eur. Assoc..

[35] Kaur S, Maslov LN, Singh N, Jaggi AS (2019). Dual role of T-type calcium channels in anxiety-related behavior. J. Basic Clin. Physiol. Pharmacol..

[36] Maeta K (2018). Comprehensive behavioral analysis of mice deficient in Rapgef2 and Rapgef6, a subfamily of guanine nucleotide exchange factors for Rap small GTPases possessing the Ras/Rap-associating domain. Mol. Brain.

[37] Ranneva SV, Pavlov KS, Gromova AV, Amstislavskaya TG, Lipina TV (2017). Features of emotional and social behavioral phenotypes of calsyntenin2 knockout mice. Behav. Brain Res..

[38] Freitag S, Schachner M, Morellini F (2003). Behavioral alterations in mice deficient for the extracellular matrix glycoprotein tenascin-R. Behav. Brain Res..

[39] Elshatory Y, Gan L (2008). The LIM-homeobox gene Islet-1 is required for the development of restricted forebrain cholinergic neurons. J. Neurosci. Off. J. Soc. Neurosci..

[40] Shapiro LP, Omar MH, Koleske AJ, Gourley SL (2017). Corticosteroid-induced dendrite loss and behavioral deficiencies can be blocked by activation of Abl2/Arg kinase. Mol. Cell. Neurosci..

[41] Coba MP (2018). Dlgap1 knockout mice exhibit alterations of the postsynaptic density and selective reductions in sociability. Sci. Rep..

[42] Gutierrez, M. A., Dwyer, B. E. & Franco, S. J. Csmd2 is a synaptic transmembrane protein that interacts with PSD-95 and is required for neuronal maturation. *eNeuro*. 10.1523/eneuro.0434-18.2019. 10.1523/ENEURO.0434-18.2019 PMC6506821 31068362

[43] Kulesskaya N, Voikar V (2014). Assessment of mouse anxiety-like behavior in the light-dark box and open-field arena: role of equipment and procedure. Physiol. Behav..

[44] Shan L, Galaj E, Ma YY (2019). Nucleus accumbens shell small conductance potassium channels underlie adolescent ethanol exposure-induced anxiety. Neuropsychopharmacol. Off. Publ. Am. Coll. Neuropsychopharmacol..

[45] Julian LJ (2011). Measures of anxiety: state-trait anxiety inventory (STAI), beck anxiety inventory (BAI), and hospital anxiety and depression scale-anxiety (HADS-A). Arthr. Care Res..

[46] Fox AS (2012). Central amygdala nucleus (Ce) gene expression linked to increased trait-like Ce metabolism and anxious temperament in young primates. Proc. Natl. Acad. Sci. USA.

[47] Fox AS (2019). Dorsal amygdala neurotrophin-3 decreases anxious temperament in primates. Biol. Psychiatry..

[48] Ashbrook DG, Williams RW, Lu L, Hager R (2015). A cross-species genetic analysis identifies candidate genes for mouse anxiety and human bipolar disorder. Front. Behav. Neurosci..

[49] Kang SS (2014). Diet and exercise orthogonally alter the gut microbiome and reveal independent associations with anxiety and cognition. Mol. Neurodegener..

[50] Kleiman SC (2015). The intestinal microbiota in acute anorexia nervosa and during renourishment: relationship to depression, anxiety, and eating disorder psychopathology. Psychosom. Med..

[51] Ek WE (2015). Exploring the genetics of irritable bowel syndrome: a GWA study in the general population and replication in multinational case-control cohorts. Gut.

[52] Humbel F (2019). Association of alterations in intestinal microbiota with impaired psychological function in patients with inflammatory bowel diseases in remission. Clin. Gastroenterol. Hepatol. Off. Clin. Pract. J. Am. Gastroenterol. Assoc..

[53] Chen YH (2019). Association between fecal microbiota and generalized anxiety disorder: severity and early treatment response. J. Affect. Disord..

[54] Lyte M, Li W, Opitz N, Gaykema RP, Goehler LE (2006). Induction of anxiety-like behavior in mice during the initial stages of infection with the agent of murine colonic hyperplasia Citrobacter rodentium. Physiol. Behav..

[55] Bruch JD (2016). Intestinal infection associated with future onset of an anxiety disorder: results of a nationally representative study. Brain Behav. Immun..

[56] Igartua C (2017). Host genetic variation in mucosal immunity pathways influences the upper airway microbiome. Microbiome.

[57] Churchill GA (2004). The collaborative cross, a community resource for the genetic analysis of complex traits. Nat. Genet..

[58] Welsh CE (2012). Status and access to the collaborative cross population. Mamm. Genome.

[59] Bourin M, Hascoët M (2003). The mouse light/dark box test. Eur. J. Pharmacol..

[60] Walters, W. *et al.* Improved bacterial 16S rRNA Gene (V4 and V4-5) and fungal internal transcribed spacer marker gene primers for microbial community surveys. *mSystems.*10.1128/mSystems.00009-15 (2016). 10.1128/mSystems.00009-15 PMC5069754 27822518

[61] Zhou Q, Chipperfield H, Melton DA, Wong WH (2007). A gene regulatory network in mouse embryonic stem cells. Proc. Natl. Acad. Sci. USA.

[62] R Core Team. *R: a language and environment for statistical computing*. https://www.R-project.org (2019).

