# Supplementary figures and images for: Gut microbiome partially mediates and coordinates the effects of genetics on anxiety-like behavior in Collaborative Cross mice
Source: Sci Rep. 2021 Jan 11;11:270. doi: 10.1038/s41598-020-79538-x (PMC7801399; doi:10.1038/s41598-020-79538-x)

Figure S1

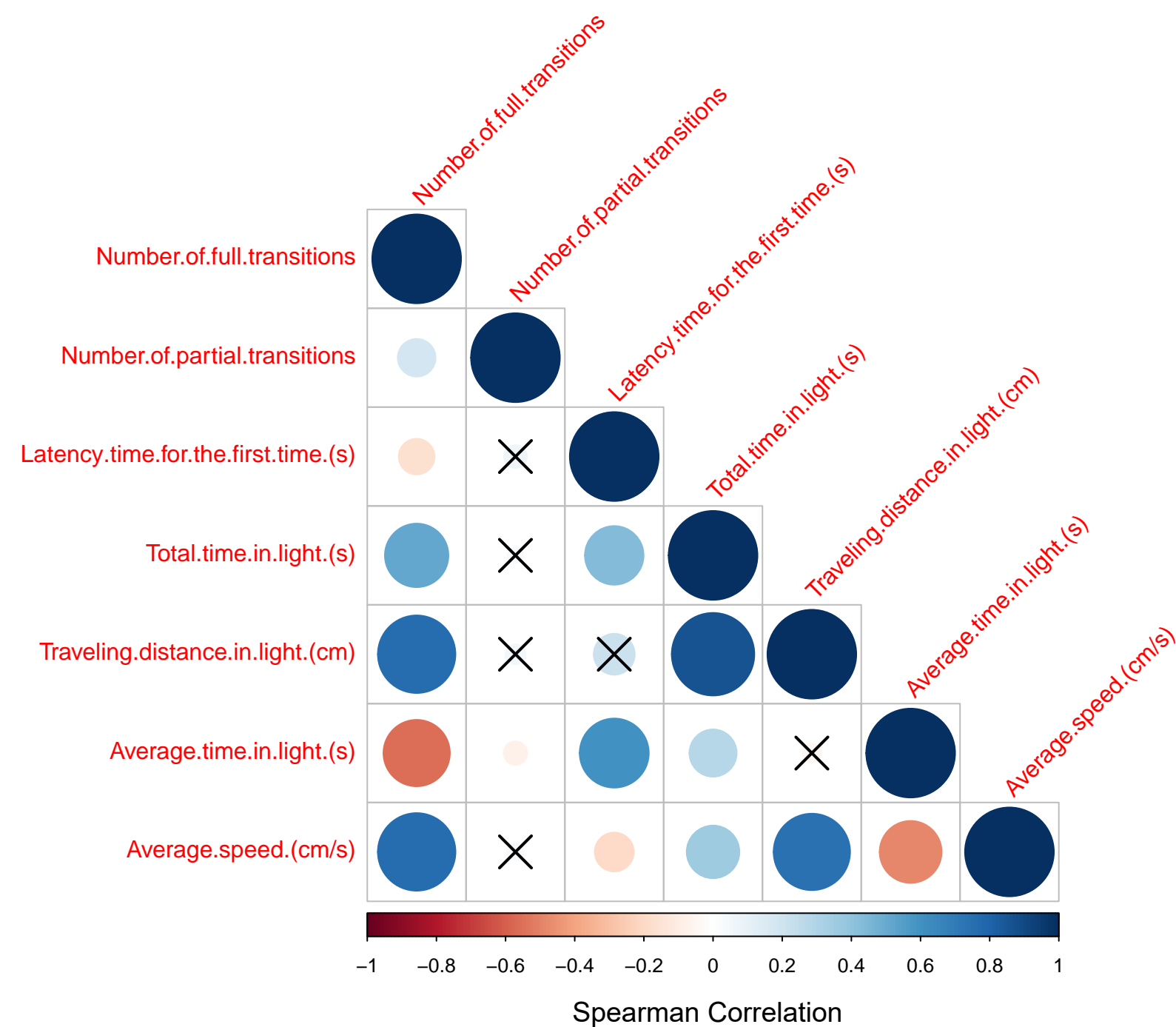

Supplement: Supplementary file 1 — Supplementary Information 1. [file 41598_2020_79538_MOESM1_ESM.pdf]

Figure S2

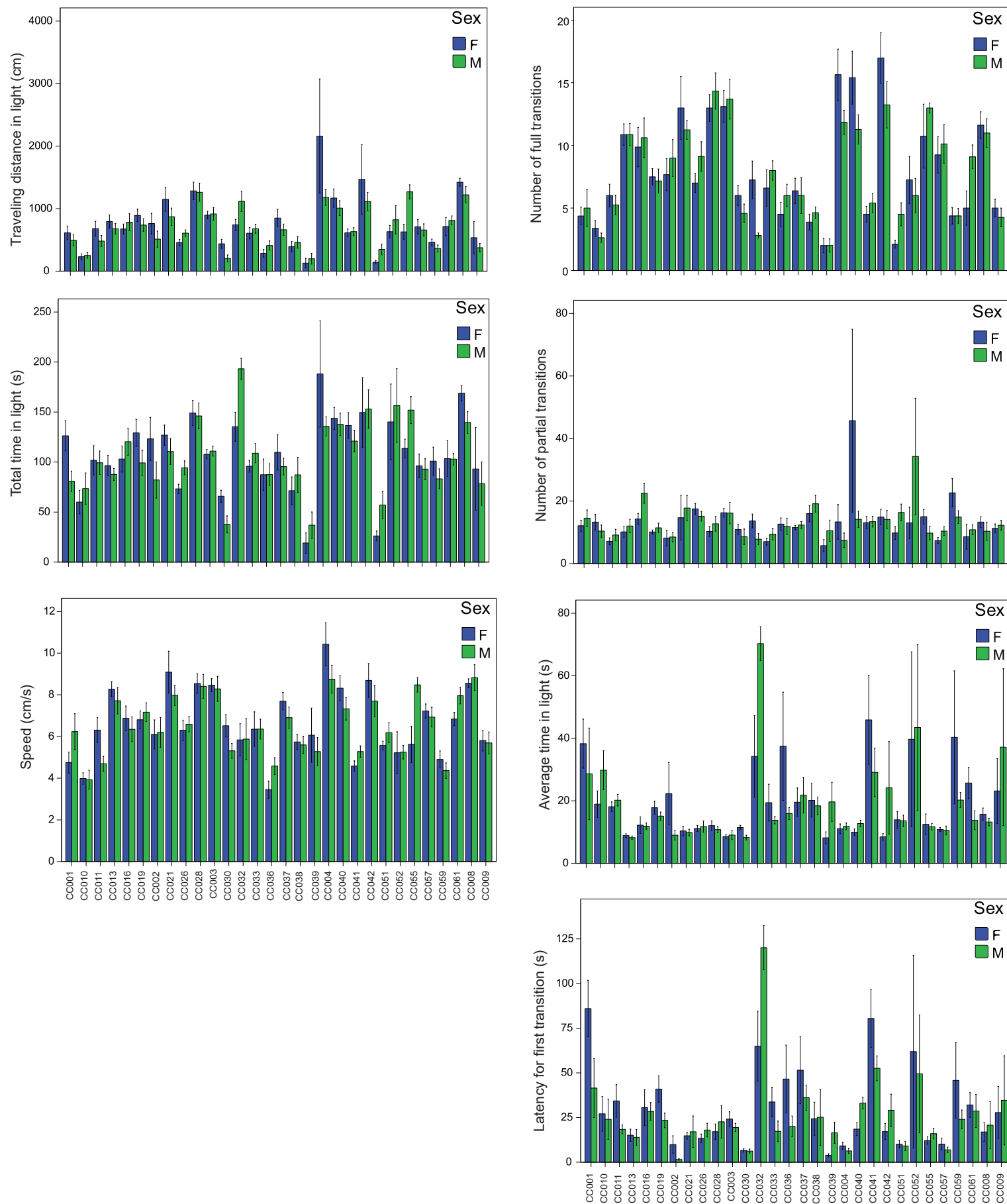

Supplement: Supplementary file 2 — Supplementary Information 2. [file 41598_2020_79538_MOESM2_ESM.pdf]

Figure S3

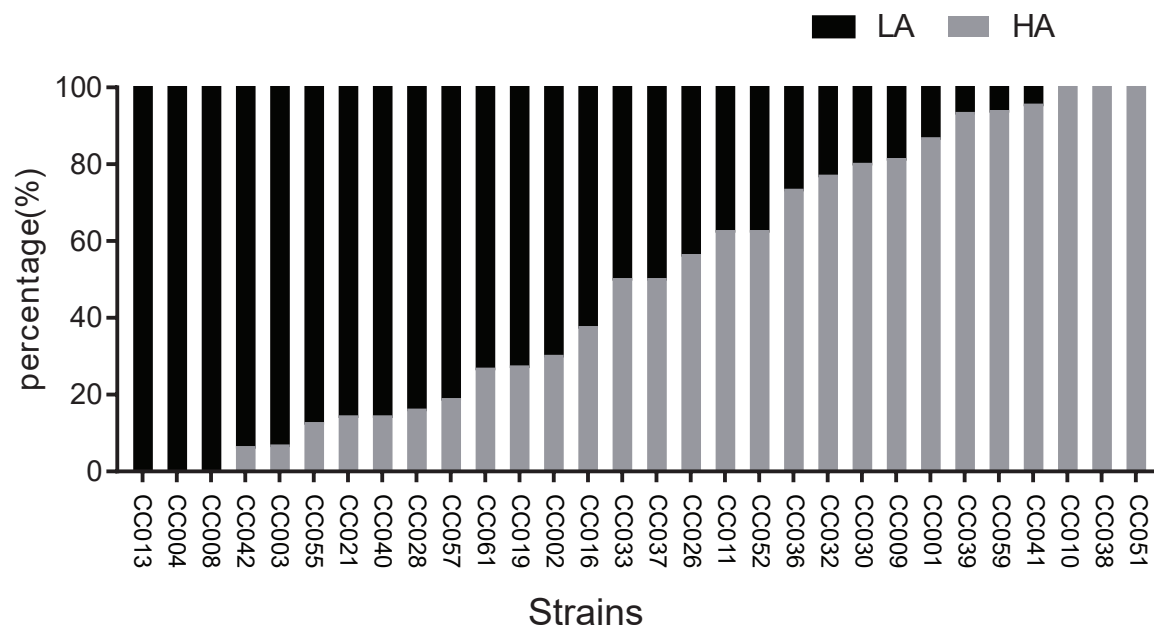

Supplement: Supplementary file 3 — Supplementary Information 3. [file 41598_2020_79538_MOESM3_ESM.pdf]
