# Supplementary material for: Gut microbiome partially mediates and coordinates the effects of genetics on anxiety-like behavior in Collaborative Cross mice
Source: Sci Rep. 2021 Jan 11;11:270. doi: 10.1038/s41598-020-79538-x (PMC7801399; doi:10.1038/s41598-020-79538-x)
Supplement: Supplementary file 4 — Supplementary Information 4. [file 41598_2020_79538_MOESM4_ESM.docx]

**Gut microbiome partially mediates and coordinates the effects of genetics on anxiety-like behavior in Collaborative Cross mice**

X. Jin^1,2,4^, Y. Zhang^3^, S. E. Celniker^2^, Y. Xia^3^, J-H. Mao^2,4^, A. M. Snijders^2,4#^, H. Chang^2,4#^

^1^ *Emergency Center, Zhongnan Hospital of Wuhan University, 169 Donghu Road, Wuhan, Hubei 430071, China*

^2^ *Biological Systems and Engineering Division, Lawrence Berkeley National Laboratory, Berkeley, California, 94720, USA.*

^3^ *State Key Laboratory of Reproductive Medicine, Center for Global Health, School of Public Health, Nanjing Medical University, Nanjing, Jiangsu, 211166, China*

^4^ *Berkeley Biomedical Data Science Center,* *Lawrence Berkeley National Laboratory, Berkeley, California, 94720, USA.*

**Supplementary Materials**

**Fig. S1 Spearman correlation between anxiety-related phenotypes.** Positive and negative correlations are displayed in blue and red color, respectively; and color intensity and the size of the circle are proportional to the correlation coefficients. Correlations that were not significant (FDR>0.05) are marked with an “x”.

**Fig. S2 Sex differences in anxiety related phenotypes across 30 CC strains.** Average measurements of seven anxiety-related phenotypes across CC strains separated by sex. Bars indicate the mean measurement and error bars indicate standard error.

**Fig. S3 Distribution of high anxiety and low anxiety mice across 30 CC strains.** Black bars indicate proportion of mice within each strain classified as low-anxiety. Gray bars indicate proportion of mice within each strain classified as high-anxiety.

**Table S1 Raw data of anxiety-related phenotypes and anxiety classification for all mice across 30 CC strains.**

**Table S2 Multivariate logistic regression between anxiety classification and seven anxiety-related phenotypes**

**Table S3 Significance level of association between each SNP and anxiety.**

**Table S4 Candidate genes located in genetic loci significantly associated with anxiety.**

**Table S5 Microbiome differences at the OTU level between the low and high anxiety mouse groups.**

**Table S6 P-values of the mediating effect of host genetics on anxiety by microbial families.**

**Table S7 List of genes between anxiety-related mouse genes and human genes associated with psychiatric conditions identified by GWAS**
